# The use of colchicine in PFAPA syndrome, the french experience and literature review

**DOI:** 10.1186/1546-0096-12-S1-P238

**Published:** 2014-09-17

**Authors:** Perrine Dusser, Bénédicte Neven, Véronique Hengten, Isabelle Koné-Paut

**Affiliations:** 1 CHU Kremlin Bicêtre, Le Kremlin Bicêtre, Paris, France; 2 CHU Paris - Hôpital Necker-Enfants Malades, Paris, France; 3 Centre hospitalier de Versailles - Hôpital André Mignot, Versailles, France

## Introduction

Background: PFAPA syndrome is the most frequent periodic fever syndrome in non-Mediterranean patients. The cause remains obscure but overexpression of inflammasome-related genes and increase IL-1b during attacks suggest an autoinflammatory mechanism. We wondered whether colchicine could be used as effective prophylactic treatment in PFAPA syndrome.

## Objectives

To compare 2 groups of PFAPA patients distinguished by their response to colchicine prophylaxis, and to identify the predictive factors of response to this treatment.

## Methods

We performed a retrospective, multicentric, chart review of PFAPA patients under colchicine prophylaxis. We distinguished one responder group, defined as patients, who had no more, or twice fewer crises under colchicine and another one of non-responders. Subgroup analyses were performed using the nonparametric Mann-Whitney test for the quantitative data and calculating the odds ratio and confidence interval for qualitative data. The difference between the two groups was considered significant for p value <0.05 or a confidence interval different from 1.

## Results

Twenty children, 65% of boys, were studied. Their mean age at disease onset was 2.3 ± 1.5 years. The mean duration of attacks was 3.2 ± 1.1 days (SD) (1 to 7 days) of strong fever (mean 39.9°C) with chills (30%), pharyngitis (85%), abdominal pain (75%), cervical adenitis (65%), asthenia (60%) and aphtous stomatitis (50%). Half of patients, 57% (8/14) were heterozygous in the *MEFV* gene. Nine patients were responders to colchicine. No significant differences were found between the two groups (responder and non-responder).

## Conclusion

We observed a relatively high rate of response to colchicine; however our study could not sort out the predictive factors of this effect.

## Disclosure of interest

None declared.

